# The inner nuclear membrane protein, Banf1, has an essential role in triple negative breast cancer cell proliferation and survival

**DOI:** 10.1038/s41598-025-10346-x

**Published:** 2025-07-15

**Authors:** Maddison Rose, Joshua T. Burgess, Chee Man Cheong, Iain Richard, Amila Suraweera, Mark N. Adams, Pascal H. G. Duijf, Kenneth J. O’Byrne, Derek J. Richard, Emma Bolderson

**Affiliations:** 1 Cancer Genomics Program, Centre for Genomics and Personalised Health, School of Biomedical Sciences, Faculty of Health, Queensland University of Technology, Kelvin Grove, Brisbane, Australia; 2 Medical Research Council Laboratory of Molecular Biology (MRC LMB), Cambridge Biomedical Campus, Francis Crick Ave, Trumpington, Cambridge, CB2 0QH UK; 3 Queensland University of Technology (QUT), Translational Research Institute, Woolloongabba, Brisbane, Australia; 4 Centre for Cancer Biology, University of South Australia & SA Pathology, Adelaide, SA Australia; 5 Cancer Services, Princess Alexandra Hospital, Ipswich Road, Woolloongabba, Brisbane, QLD 4102 Australia; 6 Queensland University of Technology, 60 Musk Avenue, Kelvin Grove, Brisbane, Australia

**Keywords:** Breast cancer, Nuclear envelope, Banf1, Mitosis, Cancer, Cell biology

## Abstract

Triple negative breast cancers (TNBCs) are a heterogenous subcategory of breast cancers which have significantly worse survival outcomes compared to other breast cancer subtypes. However, limited advances have been made towards a targeted TNBC therapeutic and traditional chemotherapies remain the frontline therapy. Therefore, this study aimed to examine the therapeutic potential of a novel TNBC target, Barrier-to-Autointegration Factor 1 (Banf1), including determining Banf1 expression and cellular localisation in non-malignant breast cells and a panel of TNBC cell lines. Bioinformatic analysis of patient samples demonstrated that Banf1 is overexpressed in all breast cancer stages and subtypes. Banf1 depletion inhibited proliferation and induced mitotic arrest in TNBC cells via loss of nuclear envelope integrity and aberrant nuclear morphology, inducing TNBC tumour cell-specific cell death. These findings highlight the significant overexpression and functional involvement of Banf1 in TNBC progression and suggests that it may have potential as a novel anti-cancer target, supporting further investigation.

**Supplementary Information:**

The online version contains supplementary material available at 10.1038/s41598-025-10346-x.

## Introduction

Triple negative breast cancer (TNBC) represents a heterogenic subset of breast cancers which do not express the oestrogen (ER) and progesterone (PR) receptors and lack overexpression of the human epidermal growth factor receptor 2 (HER2)^1,2^. TNBC is an aggressive, highly metastatic breast cancer subtype, which has significantly worse survival outcomes compared with other breast cancer subtypes^3–5^. For example, the 5-year survival rate of Stage IV TNBC patients was only 10.81%, compared to 33.46% for other breast cancer subtypes^6,7^. TNBC is generally less responsive to treatments versus non-TNBC tumours. For instance, in patients undergoing surgical and chemotherapeutic treatment modalities, 62.1% of TNBC patients were disease-free at a 5-year follow up, compared to 80.8% in non-TNBC patients^8^. Given the lack of hormone receptors, chemotherapeutic drugs remain the gold standard treatment modalities for TNBC^9^. These conventional therapies however, offer limited improvements in patient reported outcomes, whilst inducing substantial off-target, adverse effects which negatively affect the patient’s quality of life during treatment^10^. These effects are largely attributed to chemotherapies targeting all rapidly dividing cells, and not specifically malignant cells. Consequently, side effects including, nausea, vomiting, myelosuppression, and hair loss are frequently reported by patients undergoing these treatments^11,12^. Therefore, there is an evident need for the development of novel, targeted therapeutic approaches for TNBC to improve tumour response outcomes and limit adverse side effects experienced throughout treatment.

The nuclear envelope (NE) is a double membrane which was initially thought to act solely as a physical barrier separating the cytoplasmic and nuclear components of eukaryotic cells^13^. The NE and its encompassing proteins, are required for a diverse array of cellular functions, including cell division, cell signalling, transcription, cell cycle progression, chromosome tethering and, cell migration. Therefore, it is unsurprising that the dysregulation or mutation of NE proteins is linked to several pathologies, including cancer^9,14–16^.

Barrier-to-Autointegration Factor 1 (Banf1) is a NE localised protein, essential for preserving proper NE structural integrity and function^17^. Banf1 was initially recognised for its capacity to inhibit autointegration of retroviruses into their genome^18^. Banf1 has more recently been shown to have a comprehensive array of cellular functions, including chromatin organisation, regulation of DNA repair, gene expression, epigenetic regulation, and nuclear assembly^19–22^.

Banf1 has a role in mitotic progression, with its phosphorylation status acting as an essential regulator of NE breakdown and reassembly^23^. Unphosphorylated Banf1 binds to DNA and the NE via interaction with the Lem-D containing proteins^24^. Once phosphorylated, this binding is incompatible, and chromosomes dissociate from the nucleus during prophase to allow for NE breakdown. Conversely, Banf1 dephosphorylation is critical for post-miotic NE reformation^22,23^. Additionally, Banf1 depletion impairs S phase to G2 phase transition, suggesting a further role for Banf1 in cell division^22^. Banf1 has also been shown to participate in interphase NE rupture repair, with cytosolic Banf1 relocalisation to the rupture site being mediated by its affinity for double-stranded DNA. This results in the recruitment of several NE repair protein to the site, including Lemd2 and ESCRT III^25^. Banf1’s affinity for dsDNA acts as a defensive mechanism, inhibiting cyclic guanosine monophosphate–adenosine monophosphate synthase (cGAS) activity and subsequent stimulator of interferon genes (STING) activity, against self-dsDNA^26,27^. This ensures that post-NE rupture, immune responses are not activated against the cells own genetic material, potentially resulting in considerable genomic damage and cell death^28,29^.

We have previously identified several roles for Banf1 in the cellular response to DNA damage^19,20^. Banf1 negatively regulates PARP1 activity following oxidative stress, as Banf1 can outcompete the binding of NAD + to the NAD + binding domain of PARP1^19^. Banf1 also participates in the regulation of double-strand break repair pathway selection by suppressing DNA-PK activity to subsequently promote homologous recombination repair whilst suppressing non-homologous end joining^20^.

The initial evidence for Banf1’s role in tumourigenesis was as a potential biomarker for tumour prognosis in gastric and oesophageal cancer^21,30^. More recently, immunohistochemical analysis of TNBC patient samples has shown Banf1 overexpression in TNBC patient samples^31^. Here, we further examine Banf1’s role in TNBC, demonstrating that depletion impairs TNBC cellular proliferation and mitotic capacity, leading to cell death via a dysfunctional NE. Collectively, our findings support the potential of Banf1 as a promising target for TNBC treatment.

## Materials

### Reagents

#### Chemical reagents

All reagents were purchased from Sigma, unless otherwise stated.

#### Antibodies

Anti-Banf1 (SAB1409950, Sigma-Aldrich, 1:1000 for Immunoblotting (IB) and 1:500 for Immunofluorescence (IF)), anti-Emerin (5430, Cell Signalling Technology, 1:500 for IF), anti-b-Actin (2118 L, Cell Signalling Technology, 1:1000, IB), anti-Lamin A/C (4777 S, Cell Signalling Technology, 1:500, IF), anti-α-Tubulin (T5168, Sigma-Aldrich, 1:1000, IB and 1:500, IF), anti-β-actin (612656, BD Biosciences, 1:2000 for WB). Fluorescent secondary antibodies used were Donkey anti-Mouse 800 nm (LiCor; IRDye 800CW 926–32212, 1:5000, IB), Donkey anti-Rabbit (LiCor; IRDye 680LT 926–28023, 1:5000, IB) and Alexa Fluor 488 anti-Mouse (Cat# A32766, Molecular Probes, 1:200, IF) and 594 anti-Rabbit (Cat# A32754, Molecular Probes, 1:200, IF).

### Biological resources

#### SiRNA

Control (4390843) and Banf1 silencer select siRNAs were used (ThermoFisher) Scientific. Two uniquely targeting Banf1 siRNAs were utilised and are referred to as Banf1 siRNA #1 (s16808) and siRNA #2 (s16809). RNAiMax (Invitrogen) was used to transfect siRNA.

#### Cell lines

BT549, Hs578T, MDA-MB-231 and MDA-MB-468 TNBC cell lines were obtained from the ATCC (Manassas, U.S.A). MCF10A cells were used as a non-malignant, breast-tissue derived control. BT549 and Hs578T cells were cultured in RPMI media, supplemented with 10% FBS. MDA-MB-231 and MDA-MB-468 cells were cultured in DMEM media, with 10% FBS. MCF10A cells were maintained in DMEM/F12, with 20% FBS, 100 ng/mL Cholera Toxin, 20ng/mL EGF and 0.01 mg/mL insulin. Cells were cultured at 37 ºC in an atmosphere of 5% CO_2_.

### Methods

#### Immunofluorescent microscopy

Immunofluorescence (IF) was performed as previously^32^. Briefly, cells were pre-treated with extraction buffer for 5 min to visualise chromatin bound protein and fixed in 4% paraformaldehyde. Cells were permeabilised for 5 min in 0.2% Triton X-100 prior to blocking for 30 min in 3% bovine serum albumin. Cells were incubated in indicated primary antibodies and Alexa-conjugated secondary antibodies for 1 h at room temperature. Cells were countered stained in Hoechst 3342 (1 µg/mL) and imaged on a DeltaVision pDV deconvolution microscope with 100x/1.42 oil objective (Applied Precision, Inc). ImageJ was utilised to assemble images. High throughput imaging was performed using the InCell Analyzer 6500 Imaging System (GE Healthcare Life Sciences). Nuclear, cytoplasmic, and cellular staining intensity was analysed using the InCell Investigator software (GE Healthcare Life Sciences) with a minimum of 200 nuclei quantified/condition.

#### Analysis of histone H3 Serine 10 phosphorylation (pH3Ser10) via Immunofluorescence

Fixation, blocking, and imaging were completed as above, and analysis of mitotic index was completed as described^33^. Mitotic capacity was calculated using CellProfiler software v4.2.5 (Broad Institute) and reported as percentage of cells positive for pH3ser10/field of view. A minimum of 200 nuclei were analysed per experiment, for three independent repeats.

#### Immunoblotting

Cells were lysed (lysis buffer: 20 mM HEPES pH7.5, 250 mM KCl, 5% glycerol, 10 mM MgCl_2_, 0.5% Triton X-100, Protease inhibitor cocktail (Roche) and phosphatase inhibitor cocktail (Cell Signalling), sonicated and cleared by centrifugation. Typically, 30 mg of protein lysate was separated on a 4–12% BOLT Bis-Tris gel (ThermoFisher Scientific) blocked in Odyssey buffer (LiCor Biosciences) and immunoblotted with the indicated antibodies. Immunoblots were imaged using an Odyssey infrared imaging system (LiCor).

#### Quantitative real time polymerase chain reaction (qRT PCR)

Quantitative Real Time Polymerase Chain Reaction was performed as described^34^. Banf1 transcript levels were normalised to transcript levels of the 7SL house keeping gene utilising the comparative (CT) method. 7SL forward primer 5′-ATCGGGTGTCCGCACTAAGTT-3, reverse primer: 5′-CAGCACGGGAGTTTTGACCT-3. Banf1 forward primer 5′-GGGAACTGAAGTTGCGGATTA-3′, reverse primer 5′-CTCTGCCACGAAGTCTCGG-3′.

#### NE localisation and morphology quantification

Immunofluorescence was conducted as above. Localisation and quantification were performed as described^35^. Briefly, a minimum of 200 cells/condition were manually determined to have Banf1 localised/not localised to the NE Nuclear roundness’ was measured using the form factor function within the InCell Investigator software (GE Healthcare Life Sciences). The area and perimeter of each cell was measured, following InCell imaging, using the automated InCell Investigator software. The nuclear form factor was then calculated using the following equation: (form factor = $\:\frac{4\:\times\:\:\pi\:\:\times\:area}{perimete{r}^{2}}$). A form factor of 1 indicates an absolutely round nucleus, while a more irregular shape is shown by values deviating from 1. Nuclear form factor is also commonly referred to as nuclear circularity or the nuclear contour ratio, this measurement was selected as the preferred measurement of nuclear circularity as it has been shown to reflect the extent of abnormality in multi-lobed nuclei more accurately than other measurements, such as solidity or eccentricity^36,37^.

Nuclear roundness was further quantified by manually determining cells to have normal/abnormal nuclear morphology based on visual observations. Following DAPI and Lamin A staining cells were visually, manually assessed for normal or abnormal nuclear morphology. Cells with nuclear blebs (protrusions of the nuclear envelope), nuclear herniations (invaginations of the nuclear envelope) and micronuclei (small extracellular nuclei) were scored as abnormal. The data represents the percentage of cells that were abnormal in each condition.

#### Micronuclei quantification

Immunofluorescent staining and imaging were conducted as earlier described. Using Hoechst 3342 staining, 200 cells/condition were manually classified to be micronuclei positive/negative based on the presence of one or more micronuclei surrounding the nucleus.

#### Quantification of NE invaginations

Immunofluorescent staining and imaging were conducted as above. Cells were stained with anti-Lamin A/C for NE visualisation, and counterstained with nuclear Hoechst 3342. 200 cells/condition were manually classified as positive/negative for NE invaginations based on Lamin A/C staining. Representative images were compiled in ImageJ and histograms were constructed for pixel intensity lines of Lamin A/C staining as prior^38^.

#### Proliferation assay

72 h following transfection, cells were seeded at sub-confluence and following adhesion, the 96-well plate was placed into an Incucyte S3 Live-Cell Imaging System (Essen Bioscience), and a cellular confluence assay was utilised to determine proliferation rates over 5 days. Proliferation curves are representative of results and area under the curve graphs represent the mean and S.D. of three independent experiments.

#### Cell viability assay

Cell viability was quantified using an Annexin V-FITC apoptosis detection kit (Enzo Life Sciences, ALX-850-020-KI02). 5 days post transfection, cells were enzymatically lifted and media containing floating cells was collected. Cells were resuspended at 1 × 10^6^ cells/mL in 488-conjugated anti-annexin (1:40) containing binding buffer. Cells were incubated for 20 min at room temperature before staining with propidium iodide (1 mg/mL). Cells were assayed using a CytoFLEX Flow Cytometer (Beckman Coulter Life Sciences) and analysed using FlowJo software. Cells without Annexin V or PI staining were determined to be viable.

#### Bioinformatic analysis

RNAseq data from the TCGA breast cancer dataset was used to assess Banf1 transcript levels across breast cancer stages and histologies compared to adjacent normal tissue^39^. The TCGA breast cancer dataset was also utilised to assess the relationship between high/low *Banf1* expression and clinical characteristics of normal-like, luminal A, luminal B, HER2 and basal breast tumours. Box plots show median expression levels with interquartile ranges and notches show the 95% confidence intervals. Statistical significance levels were determined by unpaired Mann–Whitney *U* tests.

#### Statistical analyses

Statistical analysis of the results was made using GraphPad Prism software. T-test (two-tailed) were used for statistical analysis, unless otherwise stated. Data are presented as means and standard deviation (SD) from ≥ 3 independent experiments (unless otherwise stated). Statistical significance is represented by *: P-value < 0.05; **: P-value < 0.01; *** P-value < 0.001; ****: P-value < 0.0001.

## Results

### Banf1 is overexpressed in breast tumours and TNBC cell lines

**Fig. 1:**
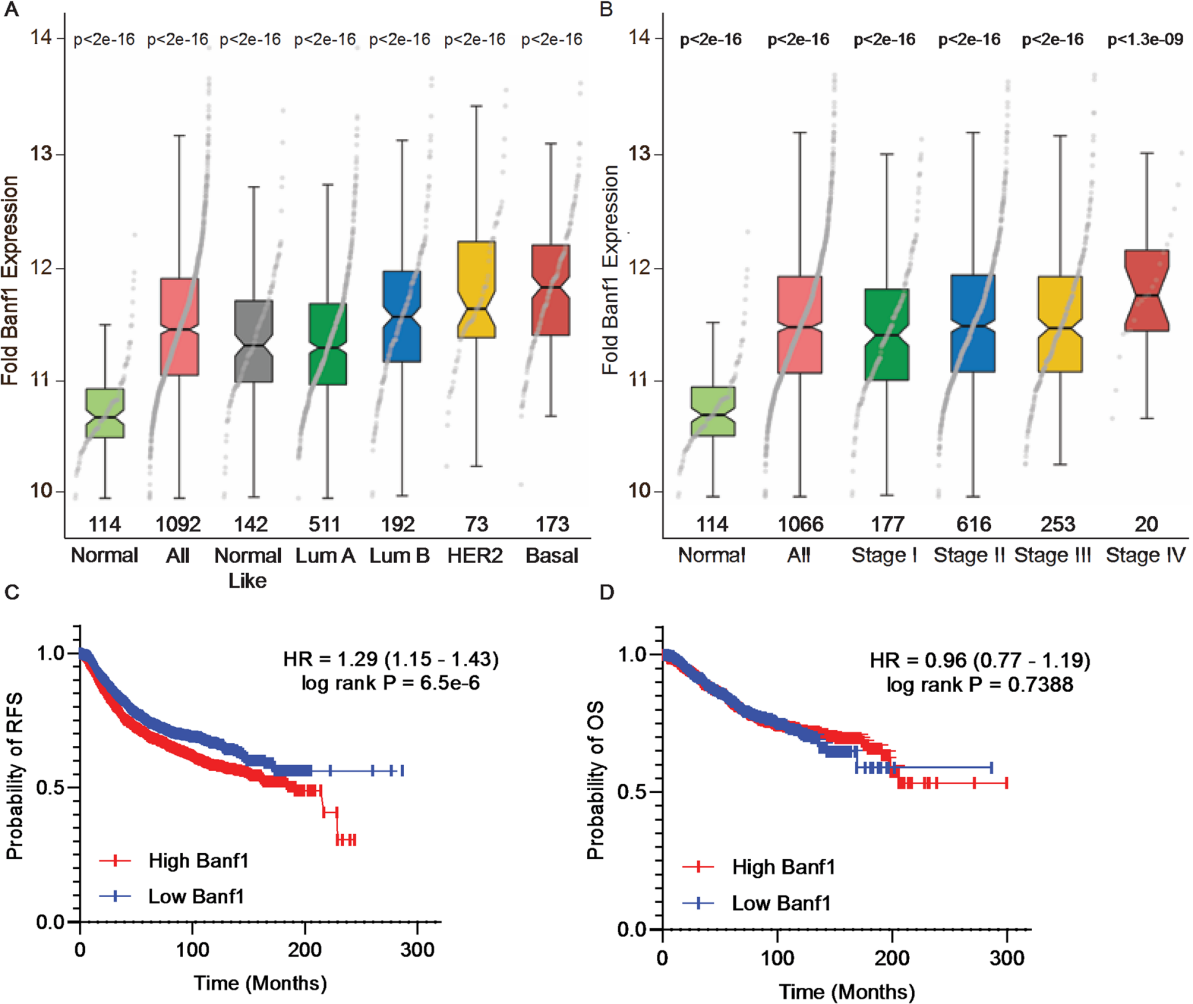
Banf1 expression is elevated in TNBC tumours. **(a – b)** Box plots comparing Banf1 gene expression levels in normal breast tissue to tumorigenic tissue from breast cancer samples within The Cancer Genome Atlas (TCGA) RNAseq dataset. P values were calculated using the unpaired Mann-Whitney U test, compared to normal tissue samples. Samples number for each group is shown on the x-axis. **(a)** Comparison of non-tumorigenic breast tissue to breast cancer stages I-IV (*n* = 1092 patients) **(b)** Comparison of non-tumorigenic breast tissue and all, normal-like, luminal A, luminal B, HER2 and basal breast carcinomas. (*n* = 1092). **(c)** Kaplan-Meier values for Banf1 mRNA expression in breast cancer patients showed high expression of Banf1 significantly decreases the probability of relapse-free survival (RFS) in breast cancer patients (*n* = 3951 patients, ****, *p* < 0.0001). Banf1 expression was divided into high or low expression based on the median expression within the dataset. Statistical significance was determined using the log-rank test in Graph Pad Prism 8. **(d)** Kaplan-Meier values for Banf1 mRNA expression in breast cancer patients showed high expression of Banf1 did not significantly decreases the probability of overall survival (OS) in breast cancer patients (ns, *p* > 0.78). Raw data was obtained for overall survival (*n* = 1402 patients) from KM plotter using the JetSet Probe Set.

To establish the prognostic implications of Banf1 expression in breast cancer patients, bioinformatic analysis was performed using The Cancer Genome Atlas (TCGA) RNAseq datasets (Fig. 1a, b)^39,40^. Consistent with earlier reports^31^*Banf1* transcripts were significantly overexpressed in all histologies and stages of breast cancer, compared to adjacent non-malignant tissue (Fig. 1a, b). *Banf1* expression levels were highest in breast cancers that are typically most aggressive, including basal-like and stage IV cancers. Given *Banf1* was overexpressed in patient data, we next investigated if Banf1 expression correlated with patient survival utilising the Kaplan-Meier dataset. Kaplan-Meier analysis of the probability of relapse free survival (RFS) in breast cancer patients was negatively correlated with *Banf1* expression, whilst there was no observable correlation between overall survival (OS) and *Banf1* expression (Fig. 1c, d).

**Fig. 2:**
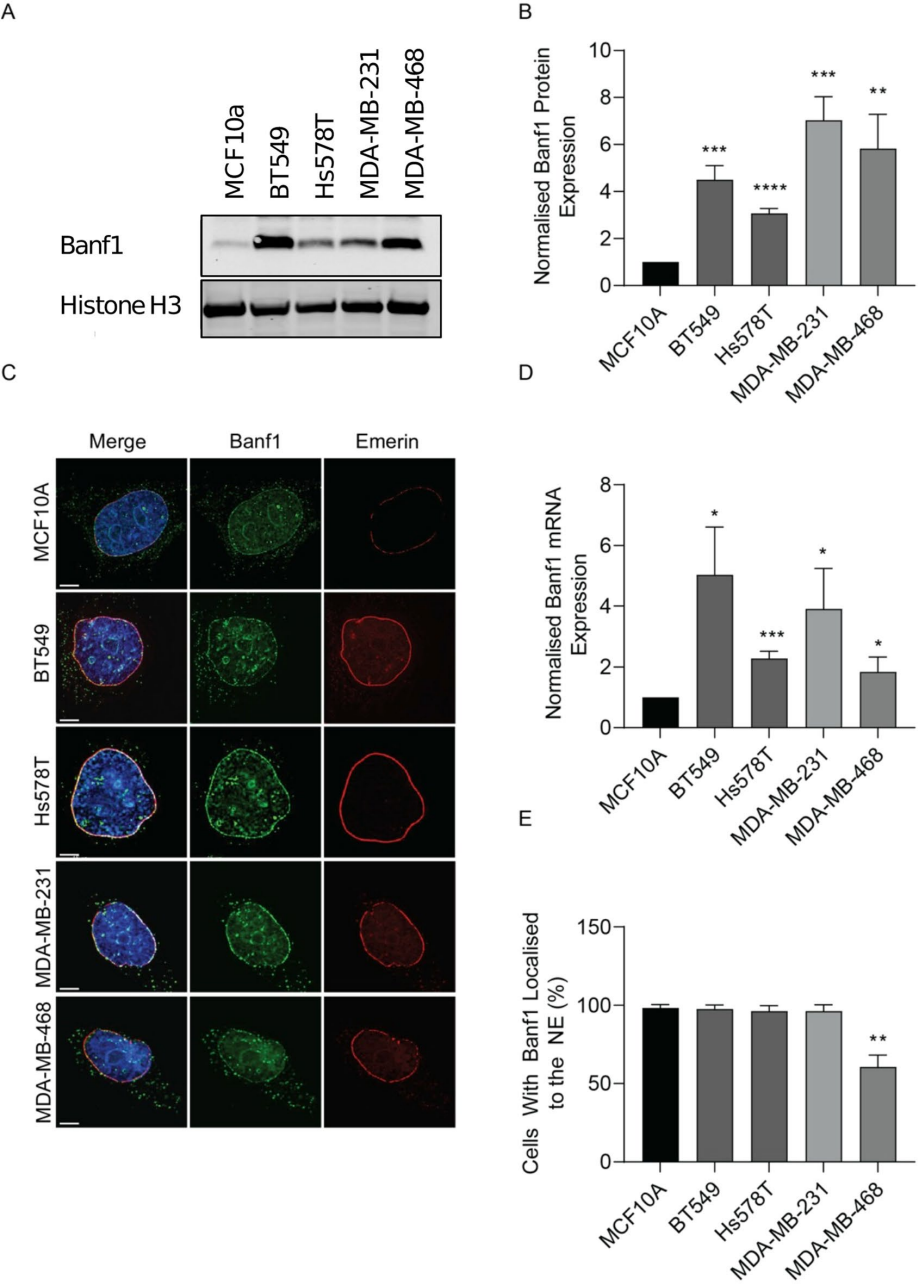
Banf1 expression is significantly elevated in TNBC cells. **(a)** Representative immunoblot of Banf1 expression in TNBC cell panel whole cell lysates compared to the control MCF10A non-malignant breast tissue cells. Blots were probed with anti-Banf1, β-Actin was utilised as a loading control and to allow for standardisation via densitometry in Image J Software. **(b)** Graph represents densitometry analysis of Banf1 expression analysed by immunoblotting in TNBC cells normalised to MCF10A cells. **(c)** Representative immunofluorescent microscopy images of MCF10A and TNBC cells. Cells were stained with anti-Banf1/anti-Emerin antibodies to visualise the NE and counterstained with Hoechst 3342. **(d)** qPCR analysis of Banf1 mRNA levels relative to the 7SL housekeeping gene compared with MCF10A cell lines and representative TNBC cell lines. Quantifications are based on 200 cells/condition per experiments. All error bars denote S.D. Scale bars = 15µM. **(e)** Quantification of the percentage of cells with NE localised Banf1. Statistical significance was calculated using unpaired t-test for **(b)**, **(c)** and **(d)**: ****, < p 0.0001, ***, < p 0.0002, **, *p* < 0.0021, *, *p* < 0.0332 (*n*=3 for all experiments).

To examine Banf1 protein levels, Banf1 mRNA and protein expression were evaluated in a TNBC cell panel and non-malignant MCF10A breast cells. Immunoblotting showed Banf1 expression at the expected size of ~ 10 kDa in cell lysates (Fig. 2a, Supp Fig. 1). Consistent with our bioinformatics analysis, densitometry analysis demonstrated significantly elevated Banf1 intensity in all tumour cell lines examined compared to non-malignant MCF10A control cells (Fig. 2b). Supporting the patient data, qPCR analysis demonstrated that *Banf1* transcript was also significantly upregulated in TNBC cell lines compared to MCF10A cells (Fig. 2c).

As Banf1 was upregulated at the RNA and protein levels in the TNBC cells examined, and abnormal NE features are characteristic of tumour cells, we next determined whether Banf1 NE localisation was disrupted in TNBC cells. High-throughput immunofluorescence imaging was undertaken to quantify the percentage of cells with NE localised Banf1. Emerin, a NE localised protein, was used as an internal control for NE visualisation. Persistent NE localised Banf1 was observed in the tumourigenic BT549, Hs578T and MDA-MB-231 cells, and MCF10A control cells. However, despite evident NE localised Emerin, only 65–70% of the MDA-MB-468 cells had detectable NE localised Banf1 (Fig. 2d, e).

### Banf1 depletion disrupts TNBC cellular morphology

**Fig. 3:**
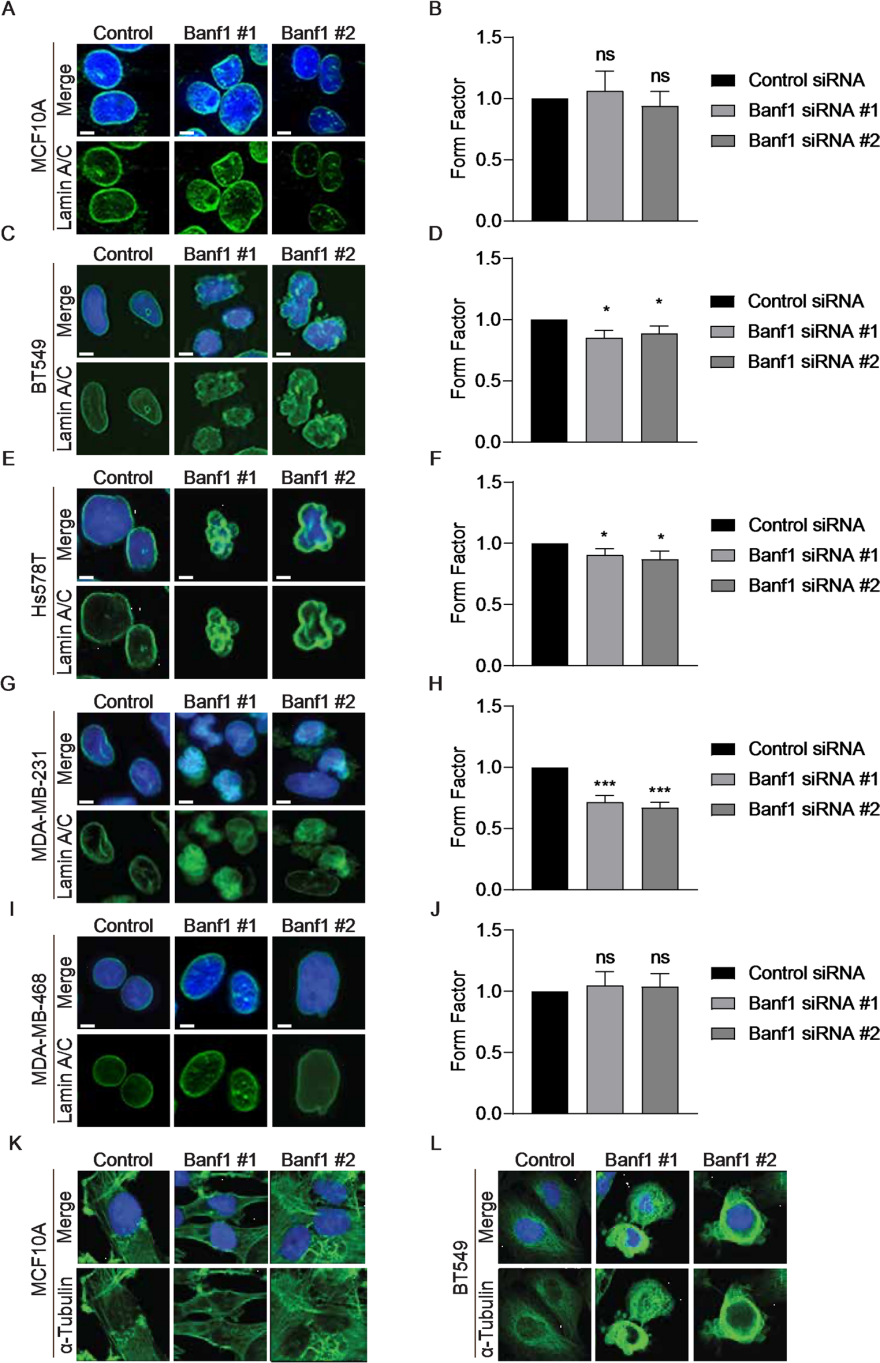
Banf1 induces aberrant NE morphology in TNBC cells. Representative immunofluorescent microscopy images of MCF10A and TNBC cells transfected with Control and Banf1 siRNAs. Cells were stained with anti-Lamin A/C to visualise the NE. Cells were counterstained with Hoechst 3342 (blue). **(a)** MCF10A **(c)** BT549 **(e)** Hs578T **(g)** MDA-MB-231 and **(i)** MDA-MB-468. Quantification of cells with aberrant nuclear morphology in Control and Banf1 transfected cells. Form Factor score of 1 = perfect round nucleus. Quantifications are based on 200 cells/condition per repeat. Error bars denote S.D. Normalised nuclear form factor values for: **(b)** MCF10A **(d)** BT549 **(f)** Hs578T **(h)** MDA-MB-231 and **(j)** MDA-MB-468. (**k - l**) Representative images of immunofluorescence staining of α-tubulin (green) in representative TNBC cell lines 72 h post-transfection with control and Banf1 siRNA. Cells were counterstained with Hoechst 3342 (blue) nuclear staining. **(k)** MCF10A **(l)** BT549. Statistical significance was calculated using unpaired t-test: ***, < p 0.0002, *, *p* < 0.0332 (*n*=3 for all experiments).

Abnormal nuclear morphology is a recognised hallmark of cancer^41^ and aberrant Banf1 expression has previously been shown to induce abnormal nuclear morphology^42^. To investigate Banf1’s role in maintaining nuclear morphology, two siRNAs (designated Banf1 siRNA #1 and #2) were used to deplete Banf1 expression. Immunofluorescent microscopy was used to analyse the effect of Banf1 depletion on nuclear morphology, using Lamin A/C to visualise the NE. Nuclear form factor values were used to assess the nuclear morphology. Briefly, a perfectly round nucleus will have a nuclear form factor value of 1, and this value decreases as the nucleus becomes less round^37^. Therefore, cells with a decreased nuclear form factor have increasingly abnormal nuclear morphology. Banf1 depletion was confirmed (Supp Fig. 2) and cells were visually categorised as having normal/abnormal nuclei as a secondary quantification method (Supp Fig. 3). Banf1 knockdown did not significantly impact the nuclear form factor of non-malignant MCF10A cells (Fig. 3a, b). However, Banf1 depletion significantly decreased nuclear form factor values across most TNBC cells, but not MDA-MB-468 cells, compared to respective controls (Fig. 3c - j), suggesting that depletion of Banf1 led to an increase in abnormal nuclear morphology in TNBC cell lines. Supporting this, there was also a significant increase in the percentage of abnormal nuclei observed following Banf1 depletion in all tumourigenic cell lines; except MDA-MB-468 cells. This is consistent with the MDA-MB-468 cell line also having Banf1 localised to the NE in a lower proportion of cells, which could potentially suggest that this cell line is less dependent upon Banf1 and therefore its depletion has less of an impact on morphology (Supp Fig. 3).

The phenotype observed following Banf1 depletion in TNBC cells was markedly similar to nuclei with NE invaginations, which are abnormal tube-like NE infoldings of the NE^38^. To further investigate this, cells were manually quantified as positive/negative for nuclear invaginations based on Lamin A/C immunofluorescent staining (Supp Fig. 4)^38^. Banf1 knockdown significantly induced NE invaginations in TNBC cells (Supp Fig. 4). This was further supported by histograms with a line drawn across nuclei, demonstrating significant Lamin A/C accumulation at proposed NE invagination sites, consistent with NE invaginations in prior studies (Supp Fig. 4a, b)^38^. Collectively, these findings suggest that Banf1 NE localisation is essential for maintaining TNBC nuclear morphology.

The relationship between nuclear and cytoskeletal morphology is well recognised and it has been established that the NE is required to maintain the structure of both cytoskeletal and nuclear components^43^. Given that Banf1 depletion induced aberrant nuclear morphology and NE disruption, it was proposed that Banf1 depletion may also impair cytoskeletal morphology. Supporting this, immunofluorescent microscopy of the structural cytoskeletal protein, alpha-tubulin, demonstrated that Banf1 depleted TNBC cells frequently display dense microtubule structures surrounding their nucleus compared to their respective controls (Fig. 3k, l, Supp Fig. 5). Thereby, suggesting that Banf1 knockdown impacts upon cytoskeletal morphology, further exacerbating the abnormal nuclear morphology of Banf1 depleted TNBC cells.

### Banf1 depletion impairs TNBC cell viability

**Fig. 4:**
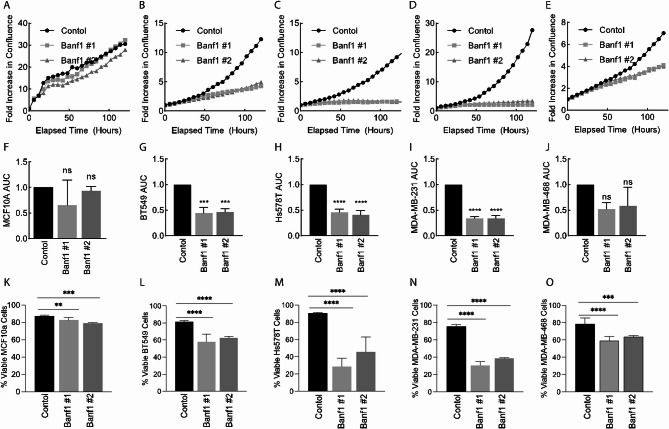
siRNA mediated Banf1 depletion inhibits TNBC cellular proliferation and induces cell death. **(a-e)**. Representative proliferation curves from 72 h post transfection with Control and Banf1 siRNAs using the Incucyte S3 live cell imaging system in MCF10A and TNBC cells. **(a)** MCF10A **(b)** BT549 **(c)** Hs578T (d) MDA-MB231 and **(e)** MDA-MB-468 **(f – j)** Relative area under the proliferation curve for **(a – e)** from independent experiments. **(k-o)** Graphs represent percent of viable cells post transfection with Control, Banf1 #1 and Banf1 #2 siRNA **(k)** MCF10A **(l)** BT549 **(m)** Hs578T **(n)** MDA-MB-231 and **(o)** MDA-MB-468. Values are normalised back to Control siRNA for respective cell lines. All graphed values represent results from individual repeats and error bars denote S.D. Statistical significance was calculated using unpaired t-test: ****, < p 0.0001, ***, < p 0.0002, **, *p* < 0.0021, *, *p* < 0.0332 (*n*=3 for all experiments).

Given Banf1 transcript and protein levels were overexpressed in TNBC and Banf1 depletion induced aberrant nuclear morphology, we next established whether Banf1 depletion impacted TNBC viability using in vitro assays. Following siRNA-transfection, proliferative capacity was evaluated over 120 h utilising an IncuCyte Analysis System. Proliferation curves (Fig. 4b – e) demonstrated that Banf1 depletion via both siRNAs drastically decreased the proliferative capacity of BT549, Hs578T and MDA-MB-231 from ~ 120 h post transfection. Area under the curve (AUC) analysis (Fig. 4f – j) further demonstrated that Banf1 depletion reduced the proliferative capacity of these cells by 60–75%. Whereas Banf1 knockdown decreased the proliferative capacity of MDA-MB-468 cells by only ~ 15%. Importantly, this was the cell line without consistent NE localised Banf1, and nuclear morphology aberrations were not induced by Banf1 depletion, suggesting that this cell line may be less dependent upon Banf1 for maintaining nuclear morphology. Banf1 depletion had minimal effect on MCF10A cell growth, decreasing the proliferative capacity by a statistically non-significant 5–35% (Fig. 4a, f). Given Banf1 upregulation was variable across the TNBC cell panel, correlation analysis of endogenous Banf1 expression and the relative decrease in AUC proliferation value was conducted and demonstrated no significant correlation between the values (Supp Fig. 6. a, b). Collectively, this suggests that the tumour cell proliferation is unlikely to be exclusively mediated by the degree of Banf1 upregulation, although further analysis using larger sample sizes is required for validation.

To establish whether cell death resulted from Banf1 depletion, an Annexin V/PI assay was conducted 5 days post-transfection (Fig. 4k – o, Supp Fig. 7). Banf1 knockdown via both siRNAs significantly decreased the percentage of viable cells in TNBC cell lines, relative to respective controls. In the non-malignant MCF10A cells there was a decrease in cell viability following Banf1 depletion; however, this was only a 6–10% decrease (Fig. 4k). Comparatively, there was a 23–29%, 32–50%, 49–60% and 1915–25% decrease in cell viability, in BT549, Hs578T, MDA-MB-231 and MDA-MB-468 cells, respectively (Fig. 4k – o). Collectively, suggesting that Banf1-depletion leads to cell death in TNBC cells.

**Fig. 5:**
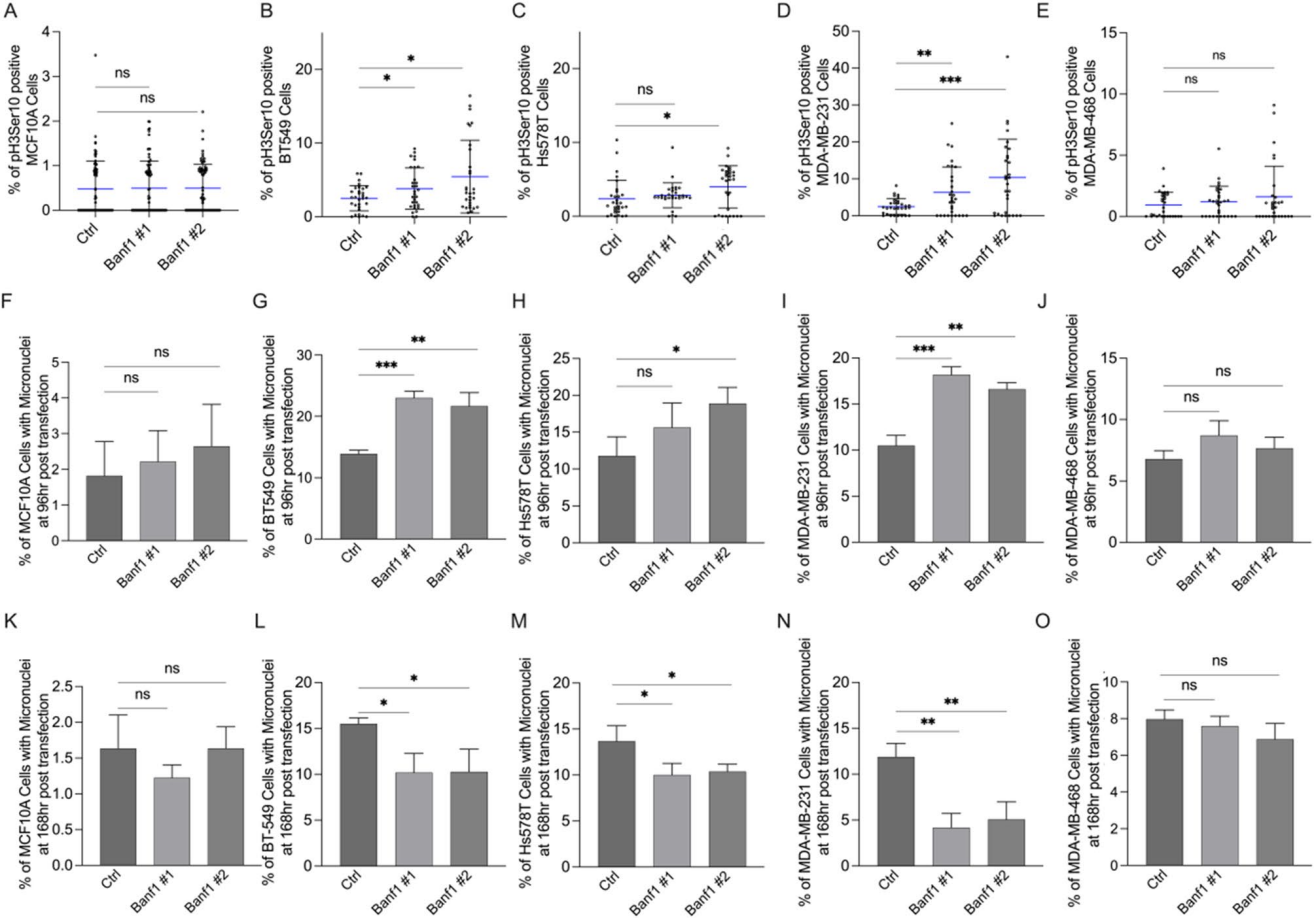
Banf1 depletion induces mitotic arrest and micronuclei formation in TNBC cells. **(a)** MCF10A **(b)** BT549, **(c)** Hs578T **(d)** MDA-MB-231 and **(e)** MDA-MB-468 cells. Percentage of micronuclei positive cells in **(f)** MCF10A, **(g)** BT549, **(h)** Hs578T, **(i)** MDA-MB-231 and **(j)** MDA-MB-468 cells 96 h following Banf1 siRNA.

Improper functioning or silencing of several NE proteins leads to mitotic arrest^44–46^. and Banf1 also has several reported roles in mitotic progression. Therefore, we proposed that Banf1 depletion induced mitotic arrest in TNBC cells. Histone H3 phosphorylation on serine 10 (pH3ser10) is a well-characterised mitosis marker, therefore a phospho-antibody that specifically recognises this modification was utilised to investigate the mitotic index (Fig. 5. a – e). In TNBC cell lines where Banf1 depletion suppressed cellular proliferation, a minimum of ~ 1.5-fold increase in the percentage of pH3ser10 positive cells was recorded, compared to respective controls (Fig. 5. a – e). However, minimal change in the prevalence of pH3ser10 positive cells was noted following Banf1 depletion in the MCF10A and MDA-MB-468 cell lines, where anti-proliferative effects were not observed.

Further supporting a role for Banf1 in mitotic functioning, micronuclei formation following improper mitosis has previously been reported in Banf1 deficient cells^47^. Given that micronuclei are known to further potentiate cell death^48^ we evaluated whether siRNA mediated Banf1 depletion altered micronuclei formation in TNBC cells (Fig. 5f – o, Supp. Figure 8a - d). Consistently, Banf1 depletion increased micronuclei prevalence in TNBC cells at 96 h post-siRNA transfection (Fig. 5f – j). However, a later 168 h time point indicates a clear reduction in the percentage of micronuclei positive cells following Banf1 depletion, suggesting that Banf1 depleted cells with micronuclei may undergo cell death or that micronuclei expulsion is occurring (Fig. 5k – o).

### Banf1 depletion induces mitotic arrest in TNBC cells

mediated depletion, compared to respective control cells. Percentage of micronuclei positive cells in **(k)** MCF10A, **(l)** BT549, **(m)** Hs578T, **(n)** MDA-MB-231 and (o) MDA-MB-468 cells 7 days post-Banf1 depletion, compared to respective control cells. Graphed values represent mean from individual repeats and error bars denote S.D. Statistical significance was calculated using unpaired t-test: ***, < p 0.0002, **, *p* < 0.0021, *, *p* < 0.0332 (*n*=3 for all experiments).

## Discussion

Here, we define a novel role for Banf1 in TNBC and as a potential anti-cancer therapeutic target. Banf1 upregulation has been demonstrated in several cancer subtypes, including breast tumours^21,30,31^. However, Banf1’s role in tumour cell survival and the therapeutic potential of targeting Banf1 has not been previously determined. Altered NE protein expression, including Lamin A/C, Ankle2 and LAP2, is reported in breast cancer models^44,49,50^. Consistently, we demonstrated *Banf1* transcript upregulation in breast cancer patient samples, and that both transcripts and protein levels were elevated in the TNBC cell lines examined, compared to non-malignant MCF10A cell line^31^.

Given frequent Banf1 overexpression in breast cancer samples, we investigated Banf1’s role in tumour cell growth. Under normal cellular conditions, Banf1 has been shown to be NE localised and is essential to maintain NE morphology^51^. Consistently, we predominately observed NE localised Banf1 in non-malignant MCF10A cells, and the majority of the TNBC cell lines examined. However, a substantial proportion of MDA-MB-468 cells lacked NE localised Banf1 whilst maintain NE localised Emerin. This suggests that these cells may not require Banf1 to maintain a structurally sound NE.

Similarly, it is reported that Banf1 suppression or aberration induces a multi-lobular or non-circular nuclear morphology^15^. We observed that Banf1 depletion induced markedly aberrant nuclear morphology in TNBC cells which possessed persistent NE localisation of Banf1, supporting the notion that Banf1’s NE localisation is essential to maintain NE integrity^47,51^. However, in MDA-MB-468 cells which did not consistently possess NE localised Banf1, there was no significant decrease in nuclear roundness or observable NE invaginations upon Banf1 depletion, suggesting this cell line is not as dependent upon Banf1 to maintain nuclear morphology. Aberrant nuclear morphology has been previously linked to heightened DNA damage, cellular senescence, inefficient mitosis and altered proliferative capacity^21,52–54^. Therefore, suggesting that the abnormal nuclear phenotype observed after Banf1 depletion may induce growth inhibiting effects on tumour cells or heighten their sensitivity to existing anti-cancer therapeutics.

Banf1 knockdown was shown to disrupt the morphology of the microtubule subunit, α-tubulin, likely due to the requirement for a structurally sound NE to link the cytoskeleton and nucleus via the SUN and KASH domain proteins^55^. Specifically, INM localised SUN proteins interact with Lamins and KASH proteins, effectively promoting KASH protein recruitment to the ONM and enabling their physically interaction with multiple cytoskeletal subunits^55,56^. Although our investigations are the first to propose a direct role for Banf1 in maintaining cytoskeletal morphology, interaction between the INM protein, Emerin, and the microtubule subunit, β-tubulin, has been reported^57^. Loss of NE organisation and stability has also been shown to induce heightened cytoskeletal tension and DNA damage response hyperactivation^58,59^. All of which have also been linked to abnormal cytoskeletal morphology and activity^60,61^. Collectively, these findings support the rationale that Banf1 depletion may disrupt α-tubulin morphology by impairing NE integrity and thus SUN-KASH protein bridges.

Proliferation, Annexin V/PI and mitotic investigations were conducted to highlight Banf1’s essential role for TNBC growth. Banf1 depletion markedly reduced proliferation and induced cell death in 3/4 TNBC cell lines examined; however, demonstrated little activity in the non-malignant MCF10A cells. Thereby, suggesting that Banf1 depletion may specifically suppress tumour cell growth.

Although our study is the first to demonstrate Banf1’s role in tumour cell proliferation, dysregulated expression of several Banf1-interacting, NE-localised proteins, including Man1 and Ankle2, have been correlated with tumour cell proliferation^62,63^. Similarly, depletion of these proteins induces death in several cellular models^44,64,65^. Thereby, indicating the importance of maintaining the NE equilibrium for cell growth regulation and providing potential insight into the mechanism by which Banf1 depletion impedes tumour cell viability.

In summary, aberrant nuclear morphology has been linked to disruption of cellular processes including migration, cell cycle progression, mitosis, and chromatin organisation^47,66–69^ highlighting the diverse mechanisms by which Banf1 depletion may impair tumour cell growth. Whilst the mechanism by which Banf1 depletion produced TNBC specific anti-proliferative effects and induced cell death hasn’t been fully established here, our data suggest it to be a multi-faceted effect.

Banf1 has several roles in maintaining mitotic functioning, largely due to Banf1’s involvement in regulating NE breakdown and reformation. For NE breakdown to occur, Banf1 undergoes VRK-mediated phosphorylation which subsequently triggers Banf1’s separation from the chromatin and Lem-D proteins^70^. Studies have shown that impairing VRK activity to inhibit Banf1 phosphorylation results in stalled nuclear breakdown and inhibits mitotic progression^24^. Similarly, PP2A- and PP4-mediated Banf1 dephosphorylation are essential for NE reformation and subsequent mitotic exit^23,71^. Considering this and our findings that Banf1 depletion induces aberrant nuclear morphology and increases pH3ser10 positive cell prevalence, it is possible that Banf1 loss may impair normal mitotic progression in TNBC cells due to NE instability. Consistent with previous studies, an initial increase in micronuclei formation following Banf1 depletion in TNBC cells was observed, previously attributed to improper mitotic progression due to loss of Banf1 mediated DNA cross bridging and chromatin compaction; therefore, downregulating chromatin mechanical stiffness^47^. However, our later time point demonstrated a marked reduction in micronuclei positive cells following Banf1 depletion. We propose this is due to elevated micronuclei ruptures in Banf1 depleted cells, induced by micronuclei NE instability due to Banf1 loss, effectively sensitising them to external stressors and promoting NE ruptures in micronuclei. Micronuclei ruptures permit cytosolic cGAS binding to genomic dsDNA, initiating a cGAS-STING dependent proinflammatory immune response through cGAMP production, subsequently leading to cell death^72^. Under normal cellular conditions, Banf1’s ability to bind dsDNA outcompetes that of cGAS and downregulates the induction of a cGAS-STING immune response^29,73^. Therefore, Banf1 depletion may upregulate cGAS binding to genomic dsDNA following micronuclei ruptures, further promoting this self-immune response and cell death. A previous study showed that combining BANF1 knockout with anti-PD-1 therapy improved therapeutic benefit as compared with anti-PD-1 alone^74^. Together with the data from the current study, this suggests that in addition to inhibiting tumour cell growth directly, inhibiting Banf1 may also synergise with immune checkpoint blockade in tumours with upregulated Banf1 levels to offer new opportunities for tumour therapy.

Given the multifaceted role of Banf1 in cellular pathways, extensive investigation is required to fully establish the role of the upregulation of Banf1 in TNBC growth and proliferation. For instance, Banf1 has multiple roles in regulating DNA repair^19,20^ and thus, it would also be prudent to further investigate whether this function contributes to the Banf1-dependent promotion of TNBC cell survival and the effects of Banf1 on the response of TNBC to DNA damage, including radio- and chemotherapy. Similarly, given Banf1’s proposed role in mitosis, Banf1 suppression may show synergy if combined with other mitosis inhibitors. Specifically, Banf1 depletion has been shown to enhance doxorubicin and paclitaxel sensitivity in MDA-MB-231 cells; however, the underlying mechanism remains unestablished^75^.

Overall, our findings demonstrate Banf1 overexpression in both breast cancer patient data and TNBC cellular models, suggesting that Banf1 may contribute to TNBC growth and survival. We also demonstrate Banf1’s essential role in maintaining TNBC nuclear morphology and proliferation, highlighting the potential for a role of Banf1, and the nuclear envelope, in tumour cell growth. In addition, Banf1 depletion induces TNBC mitotic arrest and cell death, suggesting that targeting Banf1 may improve TNBC treatments by providing a novel mechanism to specifically inhibit tumour cell growth.

## Electronic supplementary material

Below is the link to the electronic supplementary material.

Supplementary Material 1

## Data Availability

Data and materials are available upon request (E. Bolderson).

## References

[1] Garrido-Castro, A. C., Lin, N. U. & Polyak, K. Insights into molecular classifications of Triple-Negative breast cancer: improving patient selection for treatment. *Cancer Discov*. **9**, 176–198. 10.1158/2159-8290.CD-18-1177 (2019). 30679171 10.1158/2159-8290.CD-18-1177 PMC6387871

[2] Guerini-Rocco, E. et al. The repertoire of somatic genetic alterations of acinic cell carcinomas of the breast: an exploratory, hypothesis-generating study. *J. Pathol.* **237**, 166–178. 10.1002/path.4566 (2015). 26011570 10.1002/path.4566 PMC5011405

[3] Geenen, J. J. J., Linn, S. C., Beijnen, J. H. & Schellens, J. H. M. PARP inhibitors in the treatment of Triple-Negative breast Cancer. *Clin. Pharmacokinet.* **57**, 427–437. 10.1007/s40262-017-0587-4 (2018). 29063517 10.1007/s40262-017-0587-4

[4] Goto, Y. et al. Characteristics, behaviour and role of biomarkers in metastatic triple-negative breast cancer. *J. Clin. Pathol.* **73**, 147–153. 10.1136/jclinpath-2019-206078 (2020). 31563883 10.1136/jclinpath-2019-206078

[5] Sorlie, T. et al. Gene expression patterns of breast carcinomas distinguish tumor subclasses with clinical implications. *Proc. Natl. Acad. Sci. U S A*. **98**, 10869–10874. 10.1073/pnas.191367098 (2001). 11553815 10.1073/pnas.191367098 PMC58566

[6] Dent, R. et al. Triple-negative breast cancer: clinical features and patterns of recurrence. *Clin. Cancer Res.* **13**, 4429–4434. 10.1158/1078-0432.CCR-06-3045 (2007). 17671126 10.1158/1078-0432.CCR-06-3045

[7] Hsu, J. Y., Chang, C. J. & Cheng, J. S. Survival, treatment regimens and medical costs of women newly diagnosed with metastatic triple-negative breast cancer. *Sci. Rep.* **12**, 729. 10.1038/s41598-021-04316-2 (2022). 35031634 10.1038/s41598-021-04316-2 PMC8760241

[8] Gonçalves, H. Jr. et al. Survival study of Triple-Negative and Non-Triple-Negative breast Cancer in a Brazilian cohort. *Clin. Med. Insights Oncol.* **12**, 1179554918790563. 10.1177/1179554918790563 (2018). 30083066 10.1177/1179554918790563 PMC6071162

[9] Bellon, J. R., Burstein, H. J., Frank, E. S., Mittendorf, E. A. & King, T. A. Multidisciplinary considerations in the treatment of triple-negative breast cancer. *CA Cancer J. Clin.* **70**, 432–442. 10.3322/caac.21643 (2020). 32986241 10.3322/caac.21643

[10] Shen, A., Qiang, W., Wang, Y. & Chen, Y. Quality of life among breast cancer survivors with triple negative breast cancer–role of hope, self-efficacy and social support. *Eur. J. Oncol. Nurs.* **46**, 101771. 10.1016/j.ejon.2020.101771 (2020). 32506010 10.1016/j.ejon.2020.101771

[11] Haslam, I. S. & Smart, E. Chemotherapy-Induced hair loss: the use of biomarkers for predicting alopecic severity and treatment efficacy. *Biomark. Insights*. **14**, 1177271919842180. 10.1177/1177271919842180 (2019). 31037027 10.1177/1177271919842180 PMC6475836

[12] Janelsins, M. C. et al. Current pharmacotherapy for chemotherapy-induced nausea and vomiting in cancer patients. *Expert Opin. Pharmacother*. **14**, 757–766. 10.1517/14656566.2013.776541 (2013). 23496347 10.1517/14656566.2013.776541 PMC3938333

[13] Brown, R. *Observations on the organs and mode of fecundation in Orchideae and Asclepiadeae*. 1773–1858 (Taylor, 1833).

[14] Chow, K. H., Factor, R. E. & Ullman, K. S. The nuclear envelope environment and its cancer connections. *Nat. Rev. Cancer*. **12**, 196–209. 10.1038/nrc3219 (2012). 22337151 10.1038/nrc3219 PMC4338998

[15] Paquet, N. et al. Nestor-Guillermo Progeria syndrome: a biochemical insight into Barrier-to-Autointegration factor 1, Alanine 12 threonine mutation. *BMC Mol. Biol.* **15**, 27. 10.1186/s12867-014-0027-z (2014). 25495845 10.1186/s12867-014-0027-z PMC4266902

[16] Bertrand, A. T. et al. Lamin A/C assembly defects in LMNA-Congenital muscular dystrophy is responsible for the increased severity of the disease compared with Emery-Dreifuss muscular dystrophy. *Cells* **9**10.3390/cells9040844 (2020). 10.3390/cells9040844 PMC7226786 32244403

[17] Haraguchi, T. et al. Nuclear localization of barrier-to-autointegration factor is correlated with progression of S phase in human cells. *J. Cell Sci.* **120**, 1967. 10.1242/jcs.03461 (2007). 17519288 10.1242/jcs.03461

[18] Chen, H. & Engelman, A. The barrier-to-autointegration protein is a host factor for HIV type 1 integration. *Proc. Natl. Acad. Sci. U.S.A.* **95**, 15270–15274. 10.1073/pnas.95.26.15270 (1998). 9860958 10.1073/pnas.95.26.15270 PMC28032

[19] Bolderson, E. et al. Barrier-to-autointegration factor 1 (Banf1) regulates Poly [ADP-ribose] Polymerase 1 (PARP1) activity following oxidative DNA damage. *Nat. Commun.* **10**, 5501. 10.1038/s41467-019-13167-5 (2019). 31796734 10.1038/s41467-019-13167-5 PMC6890647

[20] Burgess, J. T. et al. Barrier-to-autointegration-factor (Banf1) modulates DNA double-strand break repair pathway choice via regulation of DNA-dependent kinase (DNA-PK) activity. *Nucleic Acids Res.* **49**, 3294–3307. 10.1093/nar/gkab110 (2021). 33660778 10.1093/nar/gkab110 PMC8034644

[21] Li, J. et al. Expression of VRK1 and the downstream gene BANF1 in esophageal cancer. *Biomed. Pharmacother*. **89**, 1086–1091. 10.1016/j.biopha.2017.02.095 (2017). 28298069 10.1016/j.biopha.2017.02.095

[22] Zhuang, X., Semenova, E., Maric, D. & Craigie, R. Dephosphorylation of barrier-to-autointegration factor by protein phosphatase 4 and its role in cell mitosis. *J. Biol. Chem.* **289**, 1119–1127. 10.1074/jbc.M113.492777 (2014). 24265311 10.1074/jbc.M113.492777 PMC3887179

[23] Gorjanacz, M. LEM-4 promotes rapid dephosphorylation of BAF during mitotic exit. *Nucleus* **4**, 14–17. 10.4161/nucl.22961 (2013). 23211644 10.4161/nucl.22961 PMC3585021

[24] Nichols, R. J., Wiebe, M. S. & Traktman, P. The vaccinia-related kinases phosphorylate the N’ terminus of BAF, regulating its interaction with DNA and its retention in the nucleus. *Mol. Biol. Cell.* **17**, 2451–2464. 10.1091/mbc.e05-12-1179 (2006). 16495336 10.1091/mbc.E05-12-1179 PMC1446082

[25] Halfmann, C. T. et al. Repair of nuclear ruptures requires barrier-to-autointegration factor. *J. Cell. Biol.* **218**, 2136–2149. 10.1083/jcb.201901116 (2019). 31147383 10.1083/jcb.201901116 PMC6605789

[26] Ma, H. et al. Barrier-to-Autointegration Factor 1 Protects against a Basal cGAS-STING Response. *mBio* 11, e00136-00120 (2020). 10.1128/mBio.00136-20 10.1128/mBio.00136-20 PMC7064753 32156810

[27] Wan, D., Jiang, W. & Hao, J. Research advances in how the cGAS-STING pathway controls the cellular inflammatory response. *Front. Immunol.* **11**10.3389/fimmu.2020.00615 (2020). 10.3389/fimmu.2020.00615 PMC7198750 32411126

[28] Li, X. et al. Cyclic GMP-AMP synthase is activated by double-stranded DNA-induced oligomerization. *Immunity* **39**, 1019–1031. 10.1016/j.immuni.2013.10.019 (2013). 24332030 10.1016/j.immuni.2013.10.019 PMC3886715

[29] Guey, B. et al. BAF restricts cGAS on nuclear DNA to prevent innate immune activation. *Science* **369**, 823–828. 10.1126/science.aaw6421 (2020). 32792394 10.1126/science.aaw6421

[30] Li, J. et al. Barrier-to-autointegration factor 1: A novel biomarker for gastric cancer. *Oncol. Lett.* **16**, 6488–6494. 10.3892/ol.2018.9432 (2018). https://doi.org/https://doi.org/30405787 10.3892/ol.2018.9432 PMC6202538

[31] Zhang, G. Expression and prognostic significance of BANF1 in Triple-Negative breast Cancer. *Cancer Manag Res.* **12**, 145–150. 10.2147/CMAR.S229022 (2020). 32021431 10.2147/CMAR.S229022 PMC6955598

[32] Bolderson, E. et al. Phosphorylation of Exo1 modulates homologous recombination repair of DNA double-strand breaks. *Nucleic Acids Res.* **38**, 1821–1831. 10.1093/nar/gkp1164 (2010). 20019063 10.1093/nar/gkp1164 PMC2847229

[33] Kildey, K. et al. Elevating CDCA3 levels in non-small cell lung cancer enhances sensitivity to platinum-based chemotherapy. *Commun. Biol.* **4**, 638. 10.1038/s42003-021-02136-8 (2021). 34050247 10.1038/s42003-021-02136-8 PMC8163776

[34] Paquet, N. et al. hSSB1 (NABP2/ OBFC2B) is required for the repair of 8-oxo-guanine by the hOGG1-mediated base excision repair pathway. *Nucleic Acids Res.* **43**, 8817–8829. 10.1093/nar/gkv790 (2015). 26261212 10.1093/nar/gkv790 PMC4605301

[35] Rose, M. et al. The impact of rare human variants on Barrier-To-Auto-Integration factor 1 (Banf1) structure and function. *Front. Cell. Dev. Biol.* **9**, 775441. 10.3389/fcell.2021.775441 (2021). 34820387 10.3389/fcell.2021.775441 PMC8606531

[36] Janssen, A. F. J., Breusegem, S. Y. & Larrieu, D. Current methods and pipelines for Image-Based quantitation of nuclear shape and nuclear envelope abnormalities. *Cells* **11**10.3390/cells11030347 (2022). 10.3390/cells11030347 PMC8834579 35159153

[37] Takaki, T. et al. Actomyosin drives cancer cell nuclear dysmorphia and threatens genome stability. *Nat. Commun.* **8**, 16013. 10.1038/ncomms16013 (2017). 28737169 10.1038/ncomms16013 PMC5527285

[38] Battey, E. et al. Myonuclear alterations associated with exercise are independent of age in humans. *J. Physiol.*10.1113/JP284128 (2023). 36597809 10.1113/JP284128 PMC12306410

[39] The Cancer Genome Atlas Network. Comprehensive molecular portraits of human breast tumours. *Nature* **490**, 61–70. 10.1038/nature11412 (2012). 23000897 10.1038/nature11412 PMC3465532

[40] Tomczak, K., Czerwińska, P. & Wiznerowicz, M. The Cancer genome atlas (TCGA): an immeasurable source of knowledge. *Contemp. Oncol. (Pozn)*. **19**, A68–77. 10.5114/wo.2014.47136 (2015). 25691825 10.5114/wo.2014.47136 PMC4322527

[41] de Las Heras, J. I. & Schirmer, E. C. The nuclear envelope and cancer: a diagnostic perspective and historical overview. *Adv. Exp. Med. Biol.* **773**, 5–26. 10.1007/978-1-4899-8032-8_1 (2014). 24563341 10.1007/978-1-4899-8032-8_1

[42] Molitor, T. P. & Traktman, P. Depletion of the protein kinase VRK1 disrupts nuclear envelope morphology and leads to BAF retention on mitotic chromosomes. *Mol. Biol. Cell.* **25**, 891–903. 10.1091/mbc.E13-10-0603 (2014). 24430874 10.1091/mbc.E13-10-0603 PMC3952857

[43] Starr, D. A. & Fridolfsson, H. N. Interactions between nuclei and the cytoskeleton are mediated by SUN-KASH nuclear-envelope bridges. *Annu. Rev. Cell. Dev. Biol.* **26**, 421–444. 10.1146/annurev-cellbio-100109-104037 (2010). 20507227 10.1146/annurev-cellbio-100109-104037 PMC4053175

[44] Gao, A. et al. LEM4 confers Tamoxifen resistance to breast cancer cells by activating Cyclin D-CDK4/6-Rb and ERalpha pathway. *Nat. Commun.* **9**, 4180. 10.1038/s41467-018-06309-8 (2018). 30301939 10.1038/s41467-018-06309-8 PMC6177406

[45] Lei, K. et al. Inner nuclear envelope proteins SUN1 and SUN2 play a prominent role in the DNA damage response. *Curr. Biol.* **22**, 1609–1615. 10.1016/j.cub.2012.06.043 (2012). 22863315 10.1016/j.cub.2012.06.043 PMC3466333

[46] Capo-chichi, C. D. et al. Loss of A-type lamin expression compromises nuclear envelope integrity in breast cancer. *Chin. J. Cancer*. **30**, 415–425. 10.5732/cjc.010.10566 (2011). 21627864 10.5732/cjc.010.10566 PMC3941915

[47] Samwer, M. et al. DNA Cross-Bridging shapes a single nucleus from a set of mitotic chromosomes. *Cell* **170**, 956–972. 10.1016/j.cell.2017.07.038 (2017). e923. 28841419 10.1016/j.cell.2017.07.038 PMC5638020

[48] Utani, K., Kohno, Y., Okamoto, A. & Shimizu, N. Emergence of micronuclei and their effects on the fate of cells under replication stress. *PLoS One*. **5**, e10089. 10.1371/journal.pone.0010089 (2010). 20386692 10.1371/journal.pone.0010089 PMC2851613

[49] Parise, P. et al. Lap2alpha expression is controlled by E2F and deregulated in various human tumors. *Cell. Cycle*. **5**, 1331–1341. 10.4161/cc.5.12.2833 (2006). 16760672 10.4161/cc.5.12.2833

[50] Mitchell, M. J. et al. Lamin A/C deficiency reduces Circulating tumor cell resistance to fluid shear stress. *Am. J. Physiol. Cell. Physiol.* **309**, C736–746. 10.1152/ajpcell.00050.2015 (2015). 26447202 10.1152/ajpcell.00050.2015 PMC4725441

[51] Segura-Totten, M., Kowalski, A. K., Craigie, R. & Wilson, K. L. Barrier-to-autointegration factor: major roles in chromatin decondensation and nuclear assembly. *J. Cell. Biol.* **158**, 475–485. 10.1083/jcb.200202019 (2002). 12163470 10.1083/jcb.200202019 PMC2173821

[52] Abdelfatah, N. et al. Characterization of a unique form of arrhythmic cardiomyopathy caused by recessive mutation in LEMD2. *JACC Basic. Translational Sci.* **4**, 204–221. 10.1016/j.jacbts.2018.12.001 (2019). 10.1016/j.jacbts.2018.12.001 PMC6488817 31061923

[53] Pei, S. et al. Aberrant nuclear lamina contributes to the malignancy of human gliomas. *J. Genet. Genomics*. **49**, 132–144. 10.1016/j.jgg.2021.08.013 (2022). 34530169 10.1016/j.jgg.2021.08.013

[54] Dos Santos, Á. & Toseland, C. P. Regulation of nuclear mechanics and the impact on DNA damage. *Int. J. Mol. Sci.* **22**10.3390/ijms22063178 (2021). 10.3390/ijms22063178 PMC8003950 33804722

[55] Fridkin, A., Penkner, A., Jantsch, V. & Gruenbaum, Y. SUN-domain and KASH-domain proteins during development, meiosis and disease. *Cell. Mol. Life Sci.* **66**, 1518–1533. 10.1007/s00018-008-8713-y (2009). 19125221 10.1007/s00018-008-8713-y PMC6485414

[56] Kim, D. I., Birendra, K. C. & Roux, K. J. Making the LINC: SUN and KASH protein interactions. *Biol. Chem.* **396**, 295–310. 10.1515/hsz-2014-0267 (2015). 25720065 10.1515/hsz-2014-0267 PMC4386892

[57] Salpingidou, G., Smertenko, A., Hausmanowa-Petrucewicz, I., Hussey, P. J. & Hutchison, C. J. A novel role for the nuclear membrane protein Emerin in association of the centrosome to the outer nuclear membrane. *J. Cell. Biol.* **178**, 897–904. 10.1083/jcb.200702026 (2007). 17785515 10.1083/jcb.200702026 PMC2064615

[58] Muchir, A. et al. Activation of MAPK pathways links LMNA mutations to cardiomyopathy in Emery-Dreifuss muscular dystrophy. *J. Clin. Invest.* **117**, 1282–1293. 10.1172/jci29042 (2007). 17446932 10.1172/JCI29042 PMC1849984

[59] Lei, K. et al. Inner nuclear envelope proteins SUN1 and SUN2 play a prominent role in the DNA damage response. *Curr. Biology: CB*. **22**, 1609–1615. 10.1016/j.cub.2012.06.043 (2012). 22863315 10.1016/j.cub.2012.06.043 PMC3466333

[60] Bian, H. et al. MAPK/p38 regulation of cytoskeleton rearrangement accelerates induction of macrophage activation by TLR4, but not TLR3. *Int. J. Mol. Med.* **40**, 1495–1503. 10.3892/ijmm.2017.3143 (2017). 28949380 10.3892/ijmm.2017.3143 PMC5627867

[61] Kotula, E. et al. DNA-PK target identification reveals novel links between DNA repair signaling and cytoskeletal regulation. *PLOS ONE*. **8**, e80313. 10.1371/journal.pone.0080313 (2013). 24282534 10.1371/journal.pone.0080313 PMC3840018

[62] Kong, L. et al. Lamin A/C protein is overexpressed in tissue-invading prostate cancer and promotes prostate cancer cell growth, migration and invasion through the PI3K/AKT/PTEN pathway. *Carcinogenesis* **33**, 751–759. 10.1093/carcin/bgs022 (2012). 22301279 10.1093/carcin/bgs022

[63] Markiewicz, E. et al. The inner nuclear membrane protein Emerin regulates beta-catenin activity by restricting its accumulation in the nucleus. *EMBO J.* **25**, 3275–3285. 10.1038/sj.emboj.7601230 (2006). 16858403 10.1038/sj.emboj.7601230 PMC1523183

[64] Gorjanacz, M. Nuclear assembly as a target for anti-cancer therapies. *Nucleus* **5**, 47–55. 10.4161/nucl.27928 (2014). 24637400 10.4161/nucl.27928 PMC4028355

[65] Moser, B., Basilio, J., Gotzmann, J., Brachner, A. & Foisner, R. Comparative interactome analysis of emerin, MAN1 and LEM2 reveals a unique role for LEM2 in nucleotide excision repair. *Cells* **9**10.3390/cells9020463 (2020). 10.3390/cells9020463 PMC7072835 32085595

[66] Melcon, G. et al. Loss of Emerin at the nuclear envelope disrupts the Rb1/E2F and myod pathways during muscle regeneration. *Hum. Mol. Genet.* **15**, 637–651. 10.1093/hmg/ddi479 (2006). 16403804 10.1093/hmg/ddi479

[67] Bakay, M. et al. Nuclear envelope dystrophies show a transcriptional fingerprint suggesting disruption of Rb-MyoD pathways in muscle regeneration. *Brain* **129**, 996–1013. 10.1093/brain/awl023 (2006). 16478798 10.1093/brain/awl023

[68] Karoutas, A. & Akhtar, A. Functional mechanisms and abnormalities of the nuclear lamina. *Nat. Cell. Biol.* **23**, 116–126. 10.1038/s41556-020-00630-5 (2021). 33558730 10.1038/s41556-020-00630-5

[69] Denais, C. M. et al. Nuclear envelope rupture and repair during cancer cell migration. *Science* **352**, 353–358. 10.1126/science.aad7297 (2016). 27013428 10.1126/science.aad7297 PMC4833568

[70] Gorjánácz, M. LEM-4 promotes rapid dephosphorylation of BAF during mitotic exit. *Nucleus* **4**, 14–17. 10.4161/nucl.22961 (2013). 23211644 10.4161/nucl.22961 PMC3585021

[71] Asencio, C. et al. Coordination of kinase and phosphatase activities by Lem4 enables nuclear envelope reassembly during mitosis. *Cell* **150**, 122–135. 10.1016/j.cell.2012.04.043 (2012). 22770216 10.1016/j.cell.2012.04.043

[72] Mackenzie, K. J. et al. cGAS surveillance of micronuclei links genome instability to innate immunity. *Nature* **548**, 461–465. 10.1038/nature23449 (2017). 28738408 10.1038/nature23449 PMC5870830

[73] Ma, H. et al. Barrier-to-Autointegration Factor 1 Protects against a Basal cGAS-STING Response. *mBio* 11 (2020). 10.1128/mBio.00136-20 10.1128/mBio.00136-20 PMC7064753 32156810

[74] Wang, M. et al. Inhibition of tumor intrinsic BANF1 activates antitumor immune responses via cGAS-STING and enhances the efficacy of PD-1 Blockade. *J. Immunother Cancer*. **11**10.1136/jitc-2023-007035 (2023). 10.1136/jitc-2023-007035 PMC10450060 37620043

[75] Vishnubalaji, R., Abdel-Razeq, H., Gehani, S., Albagha, O. M. E. & Alajez, N. M. Identification of a gene panel predictive of Triple-Negative breast Cancer response to neoadjuvant chemotherapy employing transcriptomic and functional validation. *Int. J. Mol. Sci.* **23**10.3390/ijms231810901 (2022). 10.3390/ijms231810901 PMC9506546 36142814

