# Supplementary material for: The inner nuclear membrane protein, Banf1, has an essential role in triple negative breast cancer cell proliferation and survival
Source: Sci Rep. 2025 Jul 15;15:25492. doi: 10.1038/s41598-025-10346-x (PMC12264273; doi:10.1038/s41598-025-10346-x)
Supplement: Supplementary file 1 — Supplementary Material 1 [file 41598_2025_10346_MOESM1_ESM.pdf]

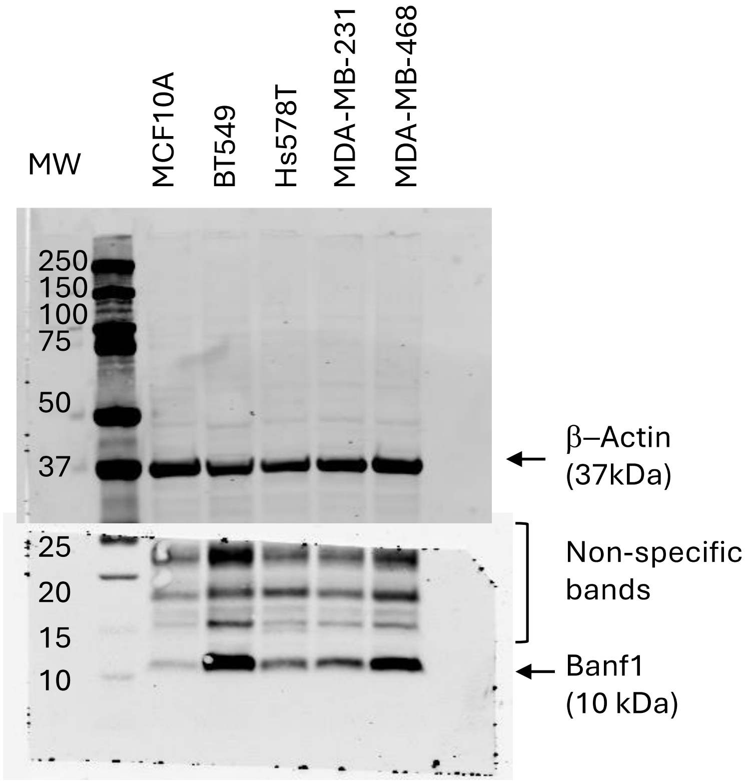

Supplementary Figure 1. The full immunoblots of Figure 2a. Representative immunoblot of Banf1 expression in TNBC cell panel whole cell lysates compared to the control MCF10A non-malignant breast tissue cells. Blots were probed with anti-Banf1 and β-Actin antibodies. β-Actin was utilised as a loading control and to allow for standardisation via densitometry in Image J Software.

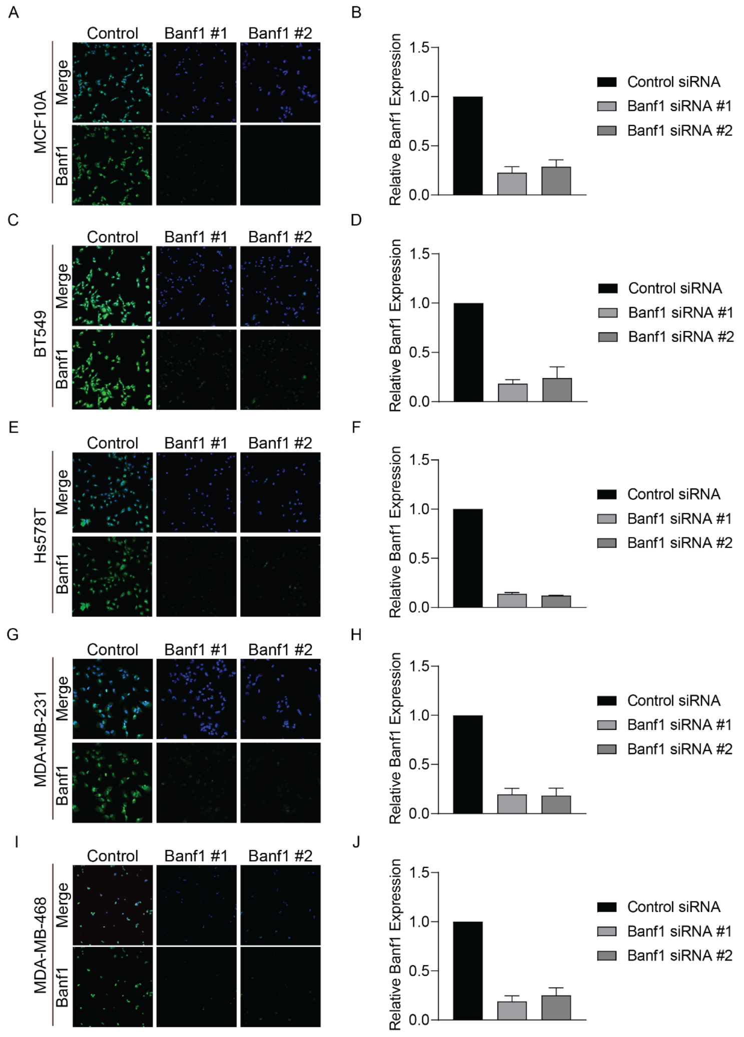

Supplementary Figure 2. Representative images of immunofluorescence staining of Banf1 (green) with anti-Banf1 antibody in TNBC cell lines 72 hours post-transfection with control or Banf1 siRNA. Cells were counterstained with Hoechst 3342 (blue) nuclear staining. (a) MCF10A (c) BT549 (e) Hs578T (g) MDA-MB-231 and (i) MDA-MB-468. Quantification of Banf1 total cell expression in control, Banf1 siRNA #1 and Banf1 siRNA #2 TNBC cells was completed using the InCell Analyser 6500 (GE Science) and corresponding analysis software. Graphed values represent results from three individual repeats and error bars denote standard deviation of the mean. (b) MCF10A (d) BT549 (f) Hs578T (h) MDA-MB-231 and (j) MDA-MB-468. (n = 3).

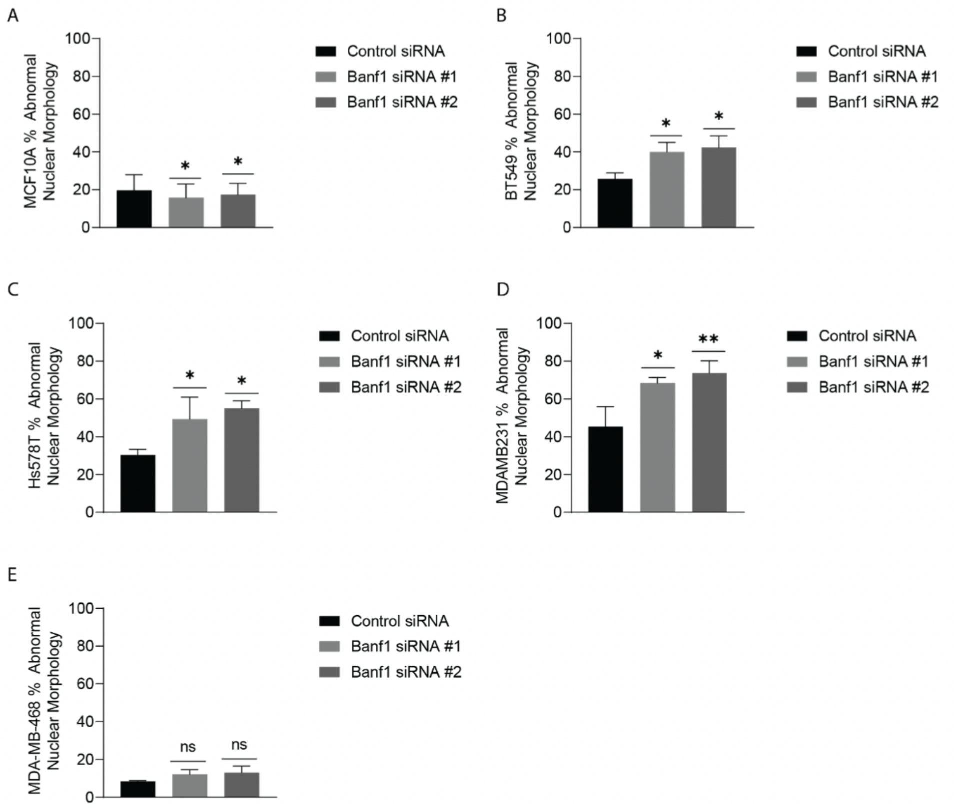

Supplementary Figure 3. Quantification of the proportion of cells where Banf1 depletion induced aberrant nuclear morphology in TNBC and MCF10A cells. Cells were visually quantified as having abnormal/normal nuclear morphology. (a) MCF10A (b) BT549 (c) Hs578T (d) MDA-MB-231 and (e) MDA-MB468. Quantifications are based on 200 cells/condition per repeat. Error bars denote standard deviation of the mean. Statistical significance was calculated using an unpaired t-test: **, $p < 0.0021$, *, $p < 0.0332$ ($n = 3$).

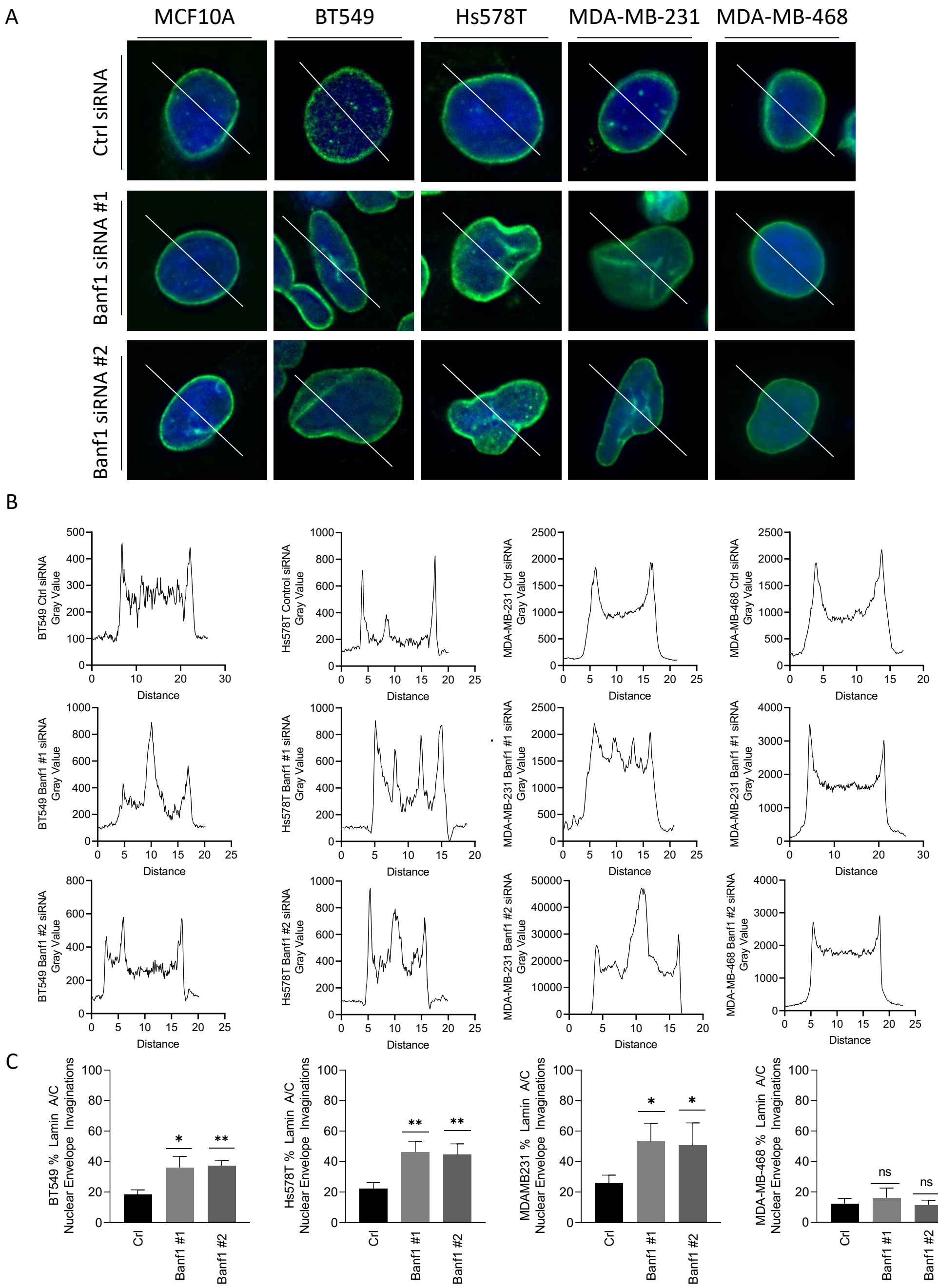

Supplementary Figure 4. Banf1 depletion induces nuclear invaginations in TNBC cells. (a) Representative images of TNBC cells following transfection with Control and Banf1 siRNA for 96 hr. Cells were stained with anti-Lamin A/C (Green) to visualise the NE. Cells were counterstained with Hoechst 3342 (blue). (b) Histograms demonstrate Lamin A/C intensity for representative control and Banf1 siRNA transfected cells for line drawn. (c) TNBC cells were visually quantified as having Lamin A/C NE invaginations Quantifications are based on a minimum of 200 cells/condition per repeat. Error bars denote standard deviation of the mean. Statistical significance was calculated using an unpaired t-test: **, $p < 0.0021$, *, $p < 0.0332$, ($n = 3$ for all experiments).

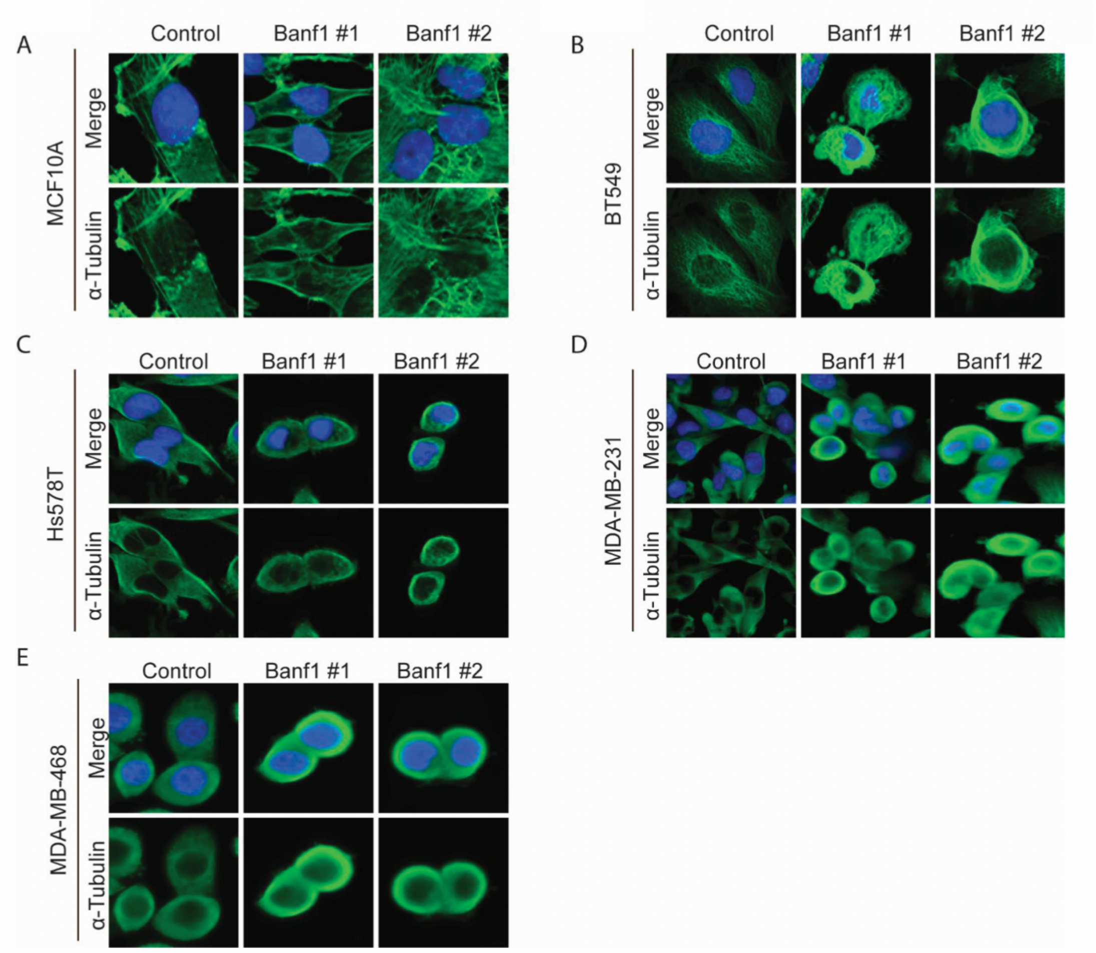

Supplementary Figure 5. siRNA mediated depletion of Banf1 impairs α-tubulin localisation in TNBC cells. (a-e) Representative images of immunofluorescence staining of α-tubulin (green) in TNBC cell lines 72 hours post-transfection with control and Banf1 siRNA. Cells were counterstained with Hoechst 3342 (blue) nuclear staining. (a) MCF10A (b) BT549 (c) Hs578T (d) MDAMB-231 and (e) MDA-MB-468 (n = 3).

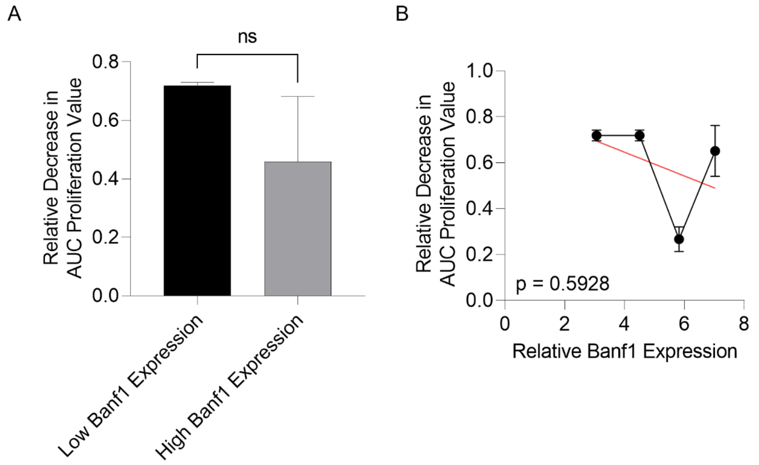

Supplementary Figure 6. Endogenous Banf1 levels do not correlate with the extent of anti-proliferative effect observed in TNBC cell lines. (a) Cell lines were categorised as having low endogenous Banf1 expression (BT549 and Hs578T) and high Banf1 expression (MDA-MB-231 and MDA-MB-468) and respective relative fold decrease in AUC values were graphed per siRNA for each cell line. (b) Correlation analysis between relative Banf1 expression and relative decrease in AUC proliferation value for BT549, Hs578T, MDA-MB-231 and MDA-MB-468 cells. Graphed values represent results from individual repeats and error bars denote standard deviation of the mean. Statistical significance was calculated using an unpaired t-test: *, $p < 0.0332$ ($n = 3$).

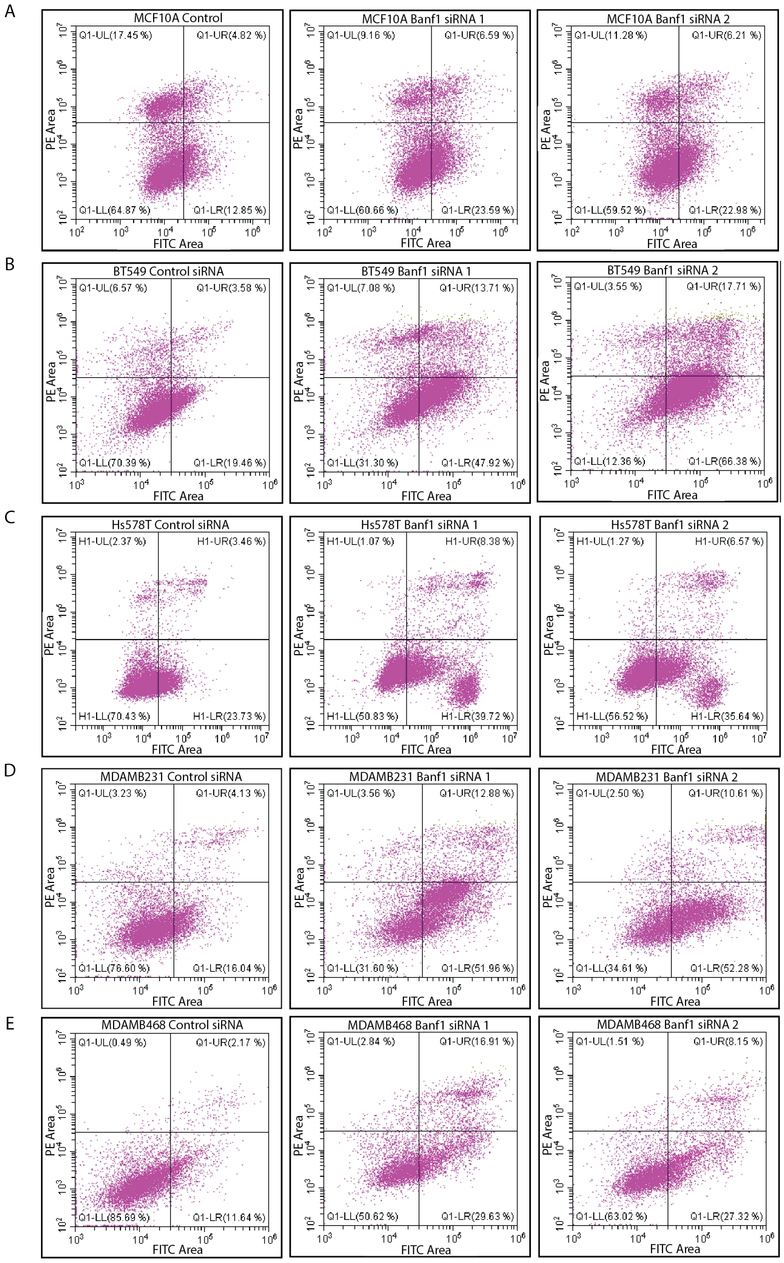

Supplementary Figure 7. Representative flow cytometry graphs for control, Banf1 siRNA #1 and Banf1 siRNA #2 transfected non-cancerous MCF10A cells and the TNBC cell lines, BT549, Hs578T, MDA-MB-231 and MDA-MB-468. Live cells were stained with propidium iodide and Annexin V 488 and assayed using a CytoFLEX flow cytometer. (Lower left): live cells, early– and late-apoptotic cells (lower right and upper left) and necrotic cells (upper left). (a) MCF10A, (b), BT549, (c) Hs578T, (d) MDA-MB-231) and (e) MDA-MB-468 (n = 3).

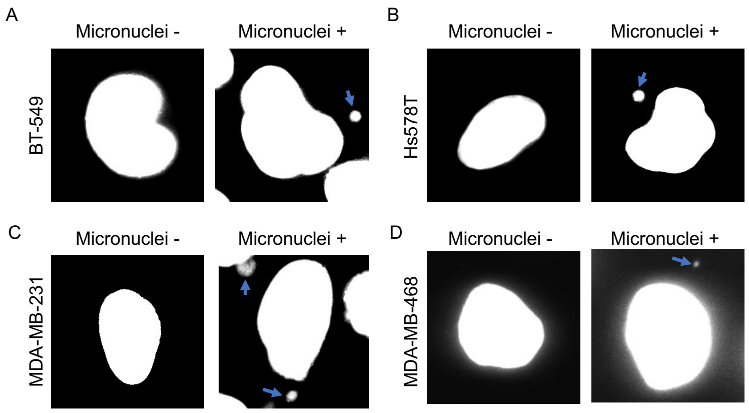

Supplementary Figure 8. siRNA mediated depletion of Banf1 induces micronuclei in TNBC cell lines. Representative images of cells positive and negative for micronuclei in (a) BT549, (b) Hs578T, (c) MDA-MB-231 and (d) MDA-MB-468 cells (n = 3).
